# Synergistic Catalytic Performance of Toluene Degradation Based on Non-Thermal Plasma and Mn/Ce-Based Bimetal-Organic Frameworks

**DOI:** 10.3390/molecules27217363

**Published:** 2022-10-29

**Authors:** Xing Rong, Qing Cao, Yan Gao, Tao Luan, Yanteng Li, Quanyou Man, Zhanchao Zhang, Baoming Chen

**Affiliations:** 1 Shandong High Speed Maintenance Group Co., Ltd., Jinan 250061, China; 2 Shandong Technology Innovation Center of Carbon Neutrality, School of Thermal Engineering, Shandong Jianzhu University, Jinan 250013, China; 3 Shandong Province Jinan Ecological and Environmental Monitoring Center, Jinan 250102, China; 4 Shandong Power Plant Thermal System Energy Saving Engineering Laboratory, School of Energy and Power Engineering, Shandong University, Jinan 250061, China

**Keywords:** metal-organic frameworks, volatile organic compounds, non-thermal plasma, bimetal, energy efficiency

## Abstract

A series of Mn/Ce-based bimetal-organic frameworks, recorded as MCDx (x = 1, 2, 4, 6), were prepared by a solvothermal synthesis method to explore their effects and performance in the synergistic catalysis of toluene under the irradiation of non-thermal plasma. The catalytic properties of different manganese loadings in MCDx for degradation of toluene were investigated. The microphysical structures of the material were analyzed by powder X-ray diffraction (PXRD), scanning electron microscopy (SEM), Fourier transform infrared spectroscopy (FTIR) and thermogravimetric analysis (TGA). The results showed that a MCDx coupling with non-thermal plasma can greatly improve the degradation efficiency, the energy efficiency and the CO_2_ selectivity, and could also significantly reduce the generation of O_3_ in the by-products. Among the test samples, MCD6 with Mn:Ce = 6:1 (molar ratio) showed the best catalytic performance and stability, exhibited toluene catalytic efficiency 95.2%, CO_2_ selectivity 84.2% and energy efficiency 5.99 g/kWh, and reduced O_3_ emission concentration 81.6%. This research provides a reference for the development and application of synergistic catalysis based on bimetal-organic frameworks and non-thermal plasma in the reduction of industrial volatile organic compounds.

## 1. Introduction

Industrial volatile organic compounds (VOCs) are one of the important sources of air pollution and have many adverse effects on human health and the ecological environment [1,2]. The methodical study and technical innovation to efficiently eliminate or reduce VOCs emissions from industrial sources has become the focus of much research, and has emerged to be the primary task of current air pollution control [3,4]. By now, various technologies have been developed to remove industrial VOCs [5,6]. According to the different technological paths, the VOCs treatments can be generally divided into two categories: the recycling method and the decomposition method. The recycling method includes adsorption, absorption, condensation and membrane separation [7,8]. The decomposition method refers to the direct destruction of the molecular structure of VOCs through chemical reactions, such as combustion, photocatalysis, thermal catalysis, biodegradation and plasma [9,10,11,12].

Traditional VOCs treatment methods have the problems of low treatment efficiency, high application cost and large energy consumption [13,14]. Plasma catalytic oxidation technology is an alternative technology for the rapid oxidation of various VOCs. A large number of active substances such as hydroxyl radicals (·OH) and reactive oxygen radicals (·O^2−^) are generated through high-voltage discharge. These active substances interact with VOCs to provoke oxidation reactions, thereby decomposing VOCs into CO_2_, H_2_O, inorganic small molecules and other substances.

This plasma processing technology can be further divided into high-temperature plasma and non-thermal plasma. In the field of VOCs exhaust treatment, non-thermal plasma (NTP) at low temperature or room temperature is the typical method [15]. Compared with other traditional VOCs treatments, NTP has the advantages of a simple treatment process and a less demanding operating environment, so it has a wide range of applications [16,17]. However, in the practical applications at present, although a single NTP is simple to operate, the process is not yet mature, the requirements for power discharge are extremely high, and the energy consumption in the process of catalyzing VOCs is huge (especially for VOCs at low or medium concentrations). Meanwhile, the catalytic process will form toxic by-products (O_3_, NO_x_, CO, etc.) causing secondary pollution problems. In order to solve the technical shortcomings of single NTP catalysis, the synergistic catalysis of VOCs by NTP and catalyst has become the main solution at this stage, which is called the plasma-catalysis system. The plasma-catalysis system can not only greatly improve the decomposition efficiency of VOCs, but more importantly, can reduce the production of toxic by-products. Therefore, the plasma-catalysis system has become one of the most promising technical methods for the treatment of industrial VOCs [18]. At present, the catalysts used in plasma-catalysis systems mainly include noble metal catalysts, non-precious metal catalysts, perovskite catalysts, and photocatalysts.

Metal-organic framework material is a new type of porous coordination polymer with periodic network structure formed by molecular self-assembly of metal ions or ion clusters and organic ligands. It has many advantages, such as high specific surface area, adjustable pore size, functional modification, and so on. It has been a research hotspot for scholars and scientific research institutions, and has been widely used in hydrogen storage, catalytic oxidation reaction, gas adsorption and medical diagnosis [19,20]. MOFs as a new catalytic material for catalytic oxidation of VOCs shortly began to attract the attention of scholars [21]. The conversion of various benzyl alcohols reached 93% and the selectivity > 99% for the hybrid material prepared by loading Pd on Cu-MOF [22]. Using MOFs material (UiO-66) supported Pd to catalyze toluene; the catalytic performance and water resistance were improved [23]. The technology of coupling MOFs with NTP catalytic elimination of VOCs is also explored [24,25]. The removal rate and CO_2_ selectivity of Mn-MIL-100 co-operated with NTP to decompose toluene were 94.7% and 44.9%, respectively [26]. MIL-101, MIL-53 and CPM-5 were used to remove toluene and isobutanol in an NTP catalytic reactor, and the removal rates were as high as 100% and 90%, which were much higher than that of the single adsorption catalytic system [27].

At present, there are relatively few studies on VOCs elimination by synergistic catalysis of NTP and MOFs; especially the experimental research using bimetallic MOFs as catalysts needs to be further analyzed. In this paper, a series of Mn/Ce-based bimetal-organic frameworks, recorded as MCDx (x = 1, 2, 4, 6), were prepared by a solvothermal synthesis method. The performance of toluene catalyzed by NTP and MCDx was studied, which provided a new perspective for the application of NTP synergistic catalysis technology.

## 2. Results and Discussion

### 2.1. Characterization and Analysis of MCDx

#### 2.1.1. Powder X-ray Diffraction (PXRD) Analysis

**Figure 1:**
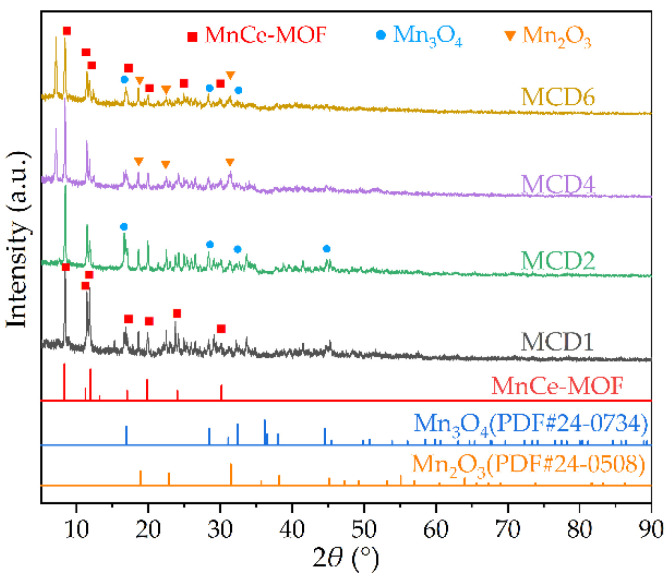
PXRD pattern of MCDx catalysts.

The PXRD analysis results of the prepared MCDx catalysts is shown in Figure 1. It can be seen from the curves that the four samples were relatively similar, with obvious diffraction peaks at around 2*θ* = 7.2°, 8.6°, 11.5°, 18.6° and 19.8°, which were consistent with the previous reports of MnCe-MOF [28,29,30], indicating MCDx series catalysts successfully prepared. At the same time, the main peak shapes of the four samples were narrow and strong, manifesting MCDx in high crystallinity. With the gradual increase of the Mn doping amount, the diffraction peak intensity of MCDx also gradually increased. When the molar ratio of Mn/Ce reached 6:1, the diffraction peak intensity of the crystal was the largest. Meanwhile, MCD6 had the highest crystallinity among the four test samples. This made clear that a large amount of Mn doping could promote the growth of MCDx crystals and impart a better crystallinity to the material. In addition, comparing the PXRD results with the PXRD standard card, it was found that the characteristic peak at 2*θ* = 23.1, 32.9° corresponded to Mn_2_O_3_ (PDF#24-0508); the (h k l) was (2 1 1) and (2 2 2), respectively [31]. The characteristic peak at 2*θ* = 18°, 28.9°, 32.3° and 44.4° matched to Mn_3_O_4_ (PDF#24-0734); the (h k l) was (1 0 1), (1 1 2), (1 0 3) and (2 2 0), respectively [32]. It was found that the peak values of Mn_2_O_3_ and Mn_3_O_4_ were low, indicating that their crystallinity in MOFs was insufficient, close to the minimum accuracy of instrument measurement and dispersed in materials. It was supposed that the valence state of Mn ions changed in different MCDx samples, which created obvious effects on the catalytic activity. In addition, some fragmentary peaks were detected in PXRD as the background noise, which did not match with other crystal materials. It was assumed that these fragmentary peaks were caused by the influence of magnetic properties of manganese oxides on the instrument. 

#### 2.1.2. Scanning Electron Microscopy (SEM) Analysis

**Figure 2:**
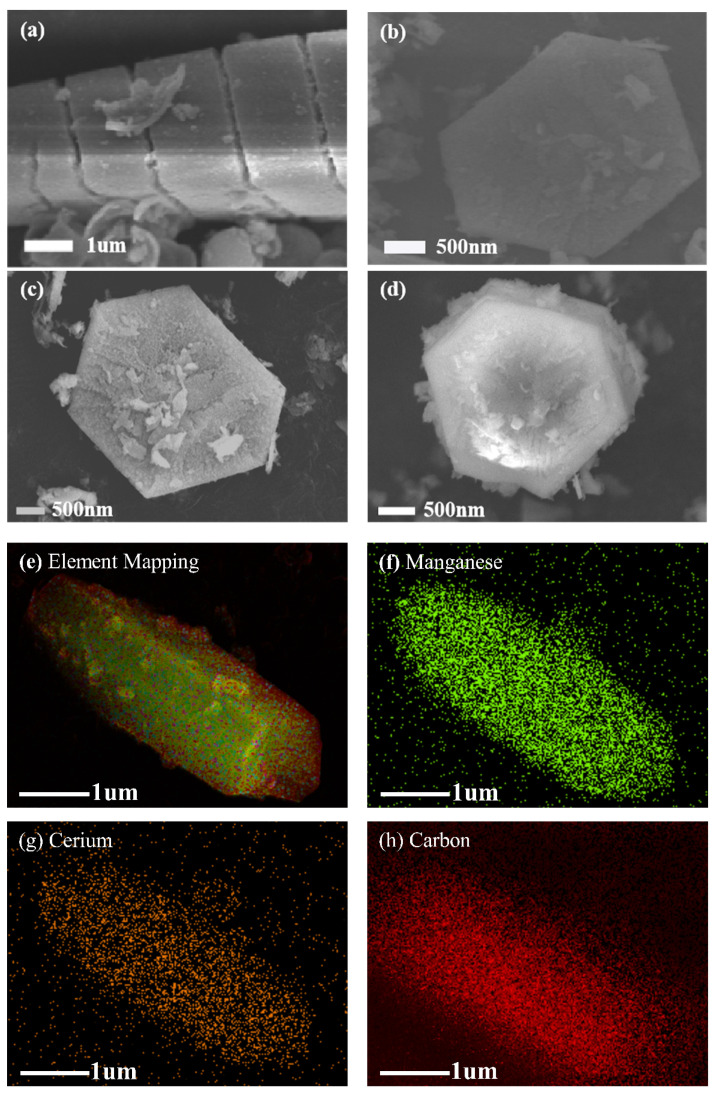

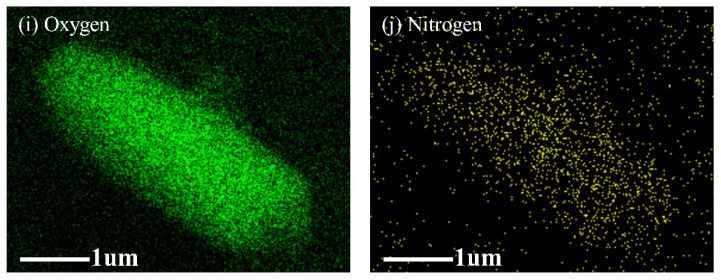
SEM image of MCDx catalysts: (**a**) MCD1; (**b**) MCD2; (**c**) MCD4; (**d**) MCD6; (**e**) EDS-Mapping of MCD6; (**f**) Element mapping of Manganese; (**g**) Element Mapping of Cerium; (**h**) Element Mapping of Carbon; (**i**) Element Mapping of Oxygen; (**j**) Element Mapping of Nitrogen.

Figure 2 presents the SEM image of MCDx (x = 1, 2, 4, 6) catalyst. This series of MCDx materials had a regular hexagonal structure with crevices on the surface. These crevices could reduce the resistance of gas diffusion, making the gas more easily adsorbed to the catalyst surface, which might be another evidence of the phase impurity, providing a large number of active sites for catalytic reactions. It can be seen from Figure 2a that the length of the cross section of a single MCDx particle size was about 2~3 μm, and a small amount of nano-sheet structure was formed on the MCDx surface. With the increase of Mn doping amount, the length of the cross section of the grain did not change greatly, but the nano-sheet structure began to appear on the surface of MCDx grain. When the Mn doping amount was further increased, the layers on the MCDx surface became more uniform and the size tended to be consistent, which covered most of the surface of the MCDx particles and filled in the crevices, as shown in Figure 2e. This shows that, in Mn/Ce bimetallic MOFs, the increase of Mn content was beneficial to the regular formation of MCDx grains. The element distribution of manganese, cerium, carbon, oxygen and nitrogen on the MCDx surface are exhibited in Figure 2f–j.

#### 2.1.3. X-ray Photoelectron Spectroscopy (XPS)

**Figure 3:**
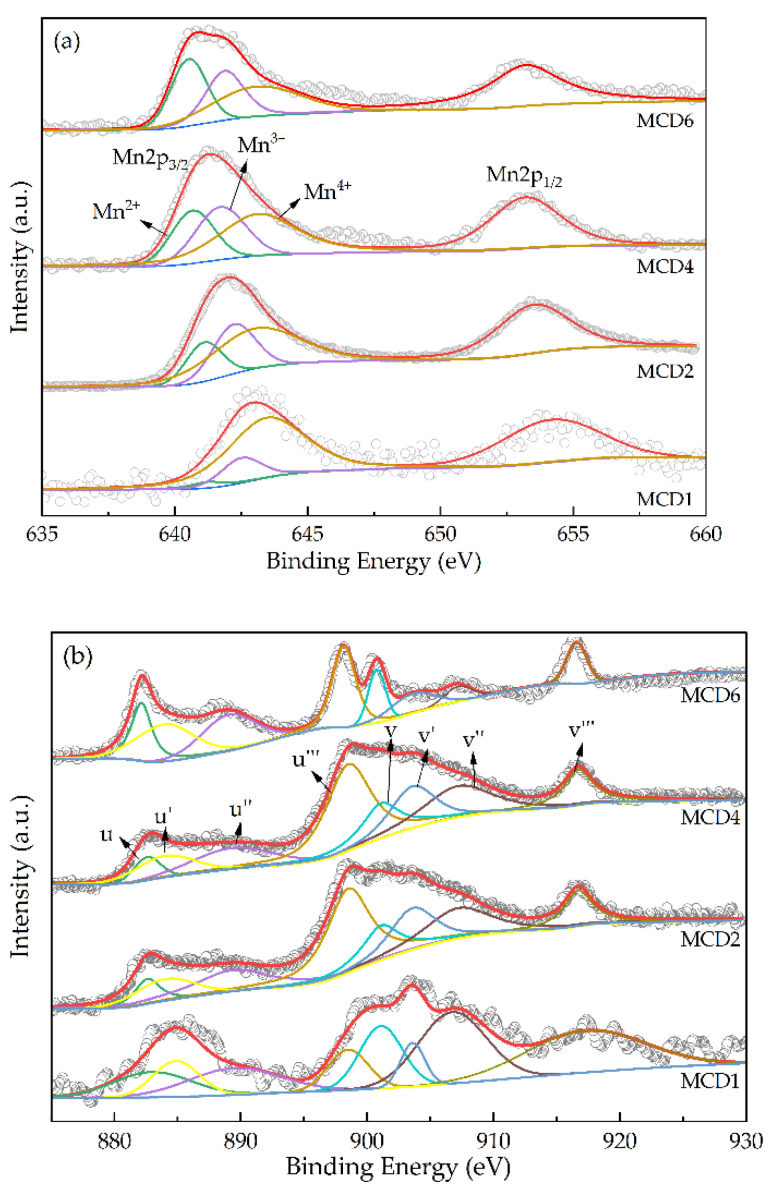
XPS spectra of the catalysts over the spectral regions: (**a**) Mn 2p; (**b**) Ce 3d.

The oxidation state and composition of the surface elements of the catalysts were analyzed by XPS, as shown in Figure 3. According to Figure 3a, Mn^2+^/Mn^3+^ and Mn^3+^/Mn^4+^ also rise with the increase of Mn content. The lower oxidation state of Mn species could improve the activity of the catalyst for the oxidation of VOCs [30]. In Figure 2b, the peaks u’ and v’ at the binding energy were mainly attributed to Ce^3+^, and the rest of the peaks were attributed to Ce^4+^. The Ce^3+^/Ce^4+^ gradually improved as the content of Mn gradually increased. The quantitative analysis of valence states of the main elements is exhibited in Table 1.

#### 2.1.4. N_2_ Adsorption–Desorption Measurement

**Figure 4:**
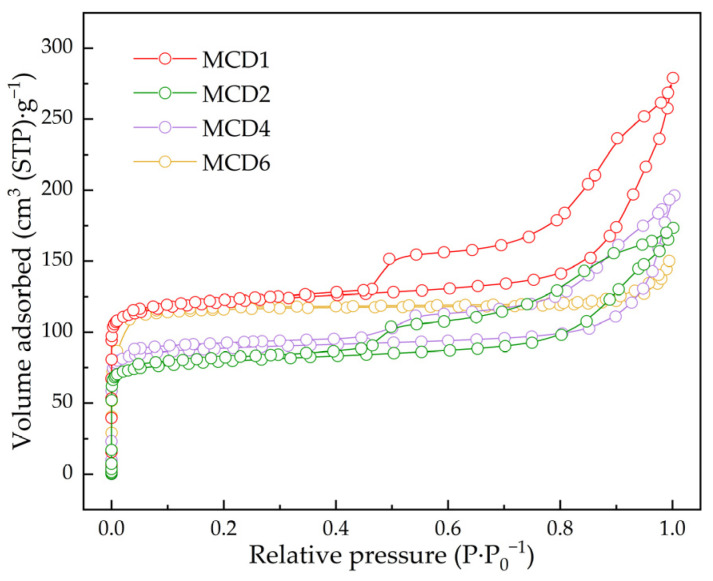
N_2_ adsorption–desorption isotherms of MCDx.

The N_2_ adsorption–desorption isotherms were the typical curves which revealed that both microporous and mesoporous structures existed in MCDx, as shown in Figure 4. The Brunauer–Emmett–Teller (BET) specific surface area of MCD1 (468.2 m^2^·g^−1^) was larger than the values of MCD2 (309.54 m^2^·g^−1^), MCD4 (350.93 m^2^·g^−1^) and MCD6 (370.70 m^2^·g^−1^). The size and shape of the adsorption hysteresis loop could be used to infer the pore structure of the adsorbent [1,14]. With the increase of manganese content, the adsorption hysteresis loop of MCDx shrunk continuously, which indicated that the proportion of mesoporous structure decreased. According to the curves, the proportion of mesoporous structure of MCD6 was smaller than that of MCD2 and MCD4, but the surface area of MCD6 was larger. This might be due to the significant increase of manganese content in MCD6, which could introduce more microporous structure, thus increasing the total specific surface area of the catalyst to a certain extent.

#### 2.1.5. Fourier Transform Infrared Spectroscopy (FTIR)

**Figure 5:**
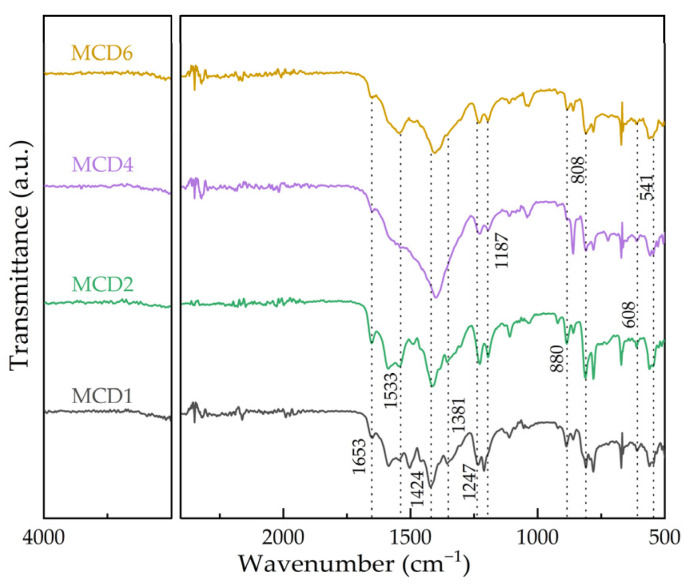
FTIR curves of MCDx catalysts.

In order to further confirm the functional group structure of the catalyst, MCDx samples were analyzed by FTIR, as shown in Figure 5. The FTIR curves of the four samples were basically the same, and each absorption peak was successfully matched with the corresponding vibration. The absorption peak at 1653 cm^−1^ was attributed to the stretching vibration peak of the hydroxyl group in organic ligand 2,5-dihydroxyterephthalic acid (DHTP) [33]. The peaks located at 1533 cm^−1^, 1424 cm^−1^, 1381 cm^−1^ and 1247 cm^−1^ could correspond to the C=O symmetric stretching, C=O asymmetric stretching, C-OH bending vibrations, and C-OH deformation vibrations of free carboxylic groups of DHTP, respectively [34,35], which exhibited an obvious shift from the typical infrared absorption spectrum, implying the deprotonation of carboxylic acid group to coordinate with manganese or cerium. The peak at 1187 cm^−1^ was indicated to be C–H in-plane of benzene ring [36,37]. The four catalysts had different wave numbers in this range, indicating that the coordination environment of the organic ligand DHTP in the MCDx structure changed at a certain extent [38]. The peaks at 608 cm^−1^ and 541 cm^−1^ correspond to the stretching vibration peaks of Mn-O and Ce-O, respectively [29], which indicated the metal ions of Mn^2+^ and Ce^3+^ were combined with the hydroxyl and carbonyl oxygen in the ligand DHTP after hydrothermal reaction to form a new chemical bond, further proving the successful synthesis of Mn/Ce bimetallic MOFs.

#### 2.1.6. Thermogravimetric Analysis (TGA)

**Figure 6:**
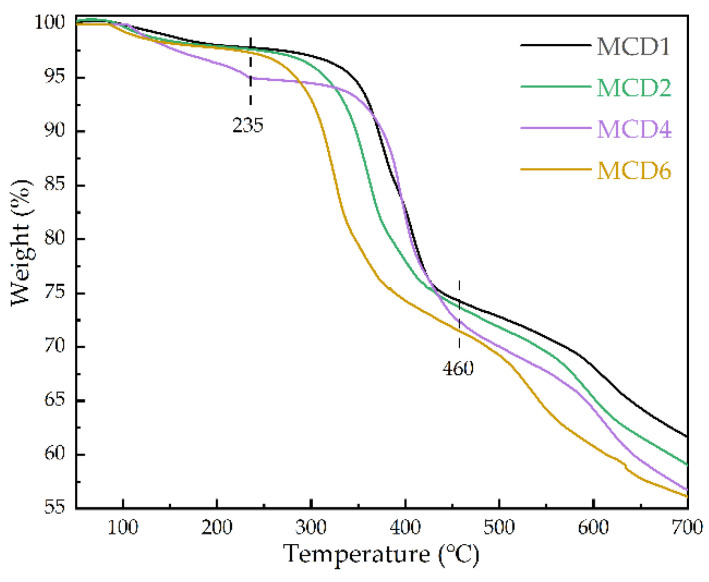
TGA test results of MCDx catalysts.

The thermal stability of MCDx catalysts were studied by thermogravimetric test in 50–700 °C with different Mn/Ce molar ratios, and the results are shown in Figure 6. It can be seen that the four samples mainly had three weightlessness stages. The first stage occurred between 50–235 °C with the weight loss rate about 5%. This stage was mainly caused by the removal of solvents (H_2_O, ethanol and DMF) adsorbed on the surface and pores of MCDx. The second stage appeared in the range of 235–460 °C, and the weight loss rate was about 20%, mainly due to the removal of coordination water molecules [39]. This stage made MCDx samples exposed a large number of Mn or Ce unsaturated metal sites and formed plenty of catalytic active sites, improving the degradation efficiency of toluene. When the temperature reached above 460 °C, the MOFs structure in the samples began to collapse and decomposed into binary metal oxide MnO_x_-CeO_2_. As it can be seen from the figure, this kind of MCDx catalyst, which could withstand roasting in range of 350–460 °C, exhibited a certain high temperature resistance.

### 2.2. Experimental Analysis of NTP Synergistic MCDx Catalyzing Toluene

#### 2.2.1. Effect of Input Power on Catalytic Performance of Toluene

1. Analysis of catalytic reactivity

**Figure 7:**
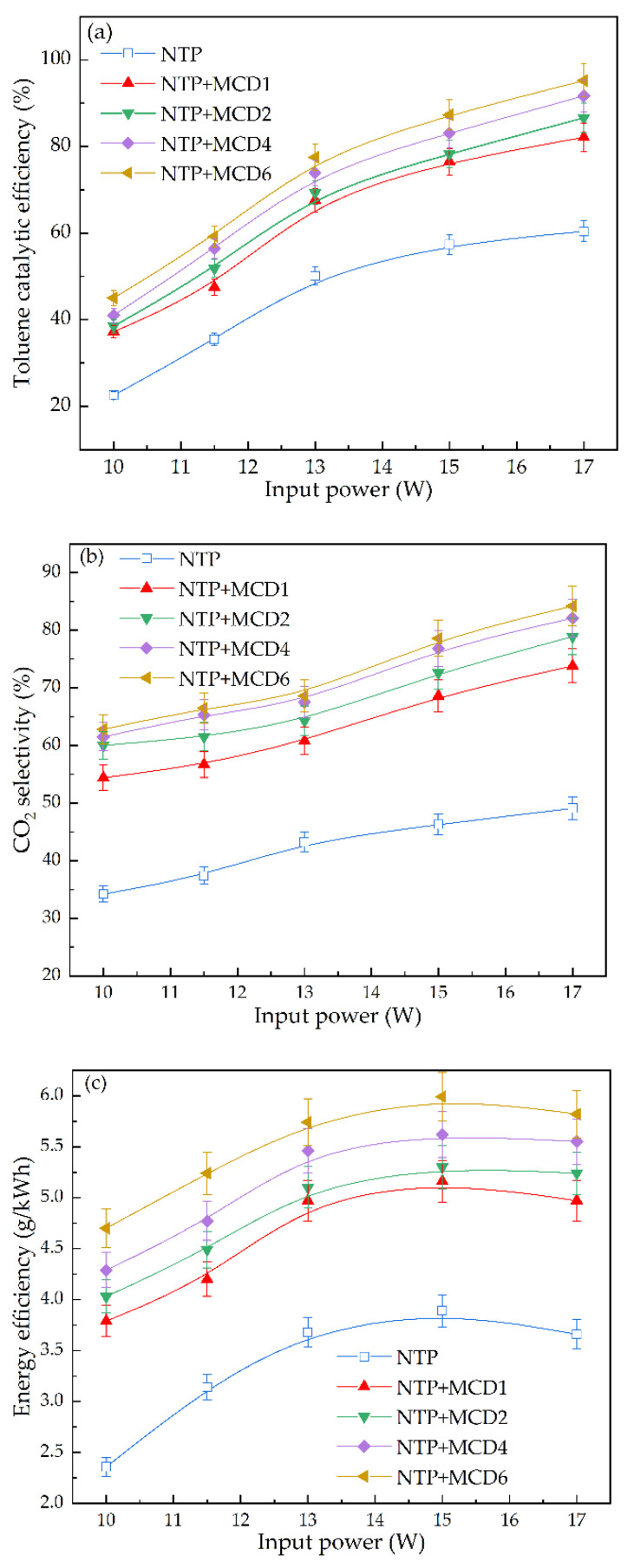
Effect of input power on synergetic catalytic performance of toluene: (**a**) catalytic reactivity; (**b**) CO_2_ selectivity; (**c**) energy efficiency.

The effect of input power on the catalysis of toluene by NTP with MCDx is shown in Figure 7. The catalytic efficiency of toluene increased with the rise of input power, which was independent of whether NTP synergistic with the catalyst. This was because the increase of input power enhanced the electric field intensity in the NTP discharge region and discharged to produce more high-energy electrons and active species, thereby promoting the degradation of toluene. According to the data shown in Figure 7a, NTP co-operate with the MCDx catalytic reaction could significantly improve the degradation efficiency of toluene. Specifically, when the input power increased from 10 W to 17 W, the toluene degradation efficiency of single NTP increased from 22.5% to 60.4%, while for the synergistic catalytic reaction of NTP and MCDx, the degradation efficiency based on MCD1, MCD2, MCD4 and MCD6 increased from 37.2%, 38.5%, 41% and 45% to 82.1%, 86.6%, 91.7% and 95.2%, respectively. This was due to the adsorption ability of MCDx-made toluene molecules and long-lived active species (such as O_3_) generated by discharge adsorbed on the surface of the catalyst [40], which further caused O_3_ decomposed on the surface of MCDx to produce strong oxidizing active oxygen and oxygen molecules, resulting in toluene molecules and intermediates’ oxidation. At the same time, the adsorption ability of MCDx also prolonged the residence time of toluene molecules in the reactor and increased the probability of collision between active species and toluene molecules, thus eliminating toluene more effectively. In addition, MCD6 co-operating NTP showed the best synergistic catalytic effect. Under optimal reaction conditions, the catalytic efficiency of MCD6 was increased by about 60% compared with single NTP, and increased by about 16%, 10% and 4% compared with MCD1, MCD2 and MCD4, respectively. The order of toluene degradation efficiency catalyzed by NTP and MCDx was: MCD6 > MCD4 > MCD2 > MCD1 > single NTP.

As shown in Figure 7b, the CO_2_ selectivity increased with enlarging input power, whether or not the catalyst was added. However, the CO_2_ selectivity of single NTP was low, and only increased from 34.2% to 49.1% as the input power increased from 10 W to 17 W. This indicated that more toluene was not completely oxidized but instead produced other organic intermediate by-products. However, after the addition of MCDx, the CO_2_ selectivity increased significantly. Specifically, the CO_2_ selectivity of MCD1, MCD2, MCD4 and MCD6 increased from 54.4%, 60%, 61.5% and 62.8% to 73.8%, 78.9%, 82.1% and 84.2%, respectively. MCD6 achieved the highest CO_2_ selectivity, which was about 71% higher than that of single NTP under optimized reaction conditions. This was due to the fact that the addition of MCDx improved the utilization of active particles such as O_3_ in the catalytic reaction, so that more active oxygen atoms produced by the decomposition of O_3_ molecules participated in the oxidation reaction of toluene, and thereby, toluene and organic intermediate products were decomposed more thoroughly, resulting in an increase of CO_2_ selectivity.

As shown in Figure 7c, no matter whether the catalyst was added, the energy efficiency increased first and then decreased with the increase of input power. This was due to the increase of input power, part of which was used to produce active species such as high-energy electrons promoting the degradation of toluene, while the other part was not involved in the reaction process but dissipated in the form of heat. Taking MCD6 as an example, when the input power increased from 10 W to 15 W, the energy efficiency increased from 4.7 g/kWh to 5.99 g/kWh, and as the input power continued to increase to 17 W, the energy efficiency decreased to 5.82 g/kWh. When the input power was 15 W, the energy efficiency of each system was the highest, as the energy efficiency of single NTP at 3.89 g/kWh, while the maximum energy efficiency of MCD1, MCD2, MCD4 and MCD6 at 5.16 g/kWh, 5.3 g/kWh, 5.62 g/kWh and 5.99 g/kWh, respectively. This indicated that the addition of MCDx improved the NTP discharge intensity and increased the amount of active particles, thereby improving energy utilization.

2. Analysis of reaction by-products

The detection and analysis of the side reaction products of toluene catalyzed by NTP and MCDx was one of the important indexes for the evaluation of the performance of the catalyst. In this experiment, N_2_ and O_2_ were used as background gases, so that by-products such as O_3_ and NO*_x_* might be produced during NTP discharge. Based on the detection of experimental instruments, it was found that there were no obvious by-products such as NO*_x_* in this experiment, and that there was almost no difference in the results of each test sample. Therefore, the concentration of by-product O_3_ was detected as the performance index. 

In the NTP synergistic catalytic system, the presence of O_3_ promoted the catalytic oxidation of toluene by MCDx, but it risked serious harm to the human body and environment. 

**Figure 8:**
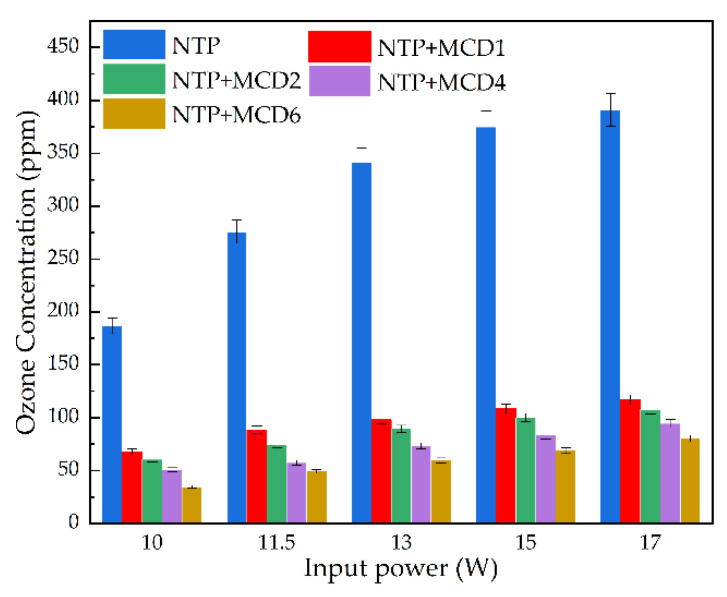
The effect of different catalysts on the O_3_ concentration in NTP-MCDx system.

It can be seen from Figure 8 that the addition of MCDx could significantly reduce the concentration of O_3_ in the exhaust gas under different discharge powers, and the utilization of O_3_ by catalysts with changed Mn doping amounts was different.

When the input power was 15 W, the O_3_ concentration in single NTP system was 374.8 ppm. For the NTP-MCDx system, the O_3_ emission concentrations of MCD1, MCD2, MCD4 and MCD6 were 108.4 ppm, 100 ppm, 83.2 ppm and 68.9 ppm, respectively. Compared with the single NTP, the purification efficiencies were increased by 57.8%, 71.1%, 73.3%, 77.8% and 81.6%, respectively. This was due to the adsorption ability of MCDx which prolonged the residence time of O_3_ molecules on the catalyst surface in the reactor, allowing more O_3_ molecules to decompose at the active sites on its surface and produce active oxygen atoms and oxygen molecules. These active particles continuously catalyze the decomposition of VOCs and accelerate the continuous consumption of O_3_ molecules, thereby affecting the O_3_ concentration in the gas at the outlet and further improving the catalytic reaction efficiency of toluene. The reaction process was shown in Equations (1)–(3):

O_3_ + * → O_2_ + O* (1)

O_3_ + O* → O_2_ + O_2_* (2)

O_2_* → O_2_ + * (3)

where * represented all active sites that can react on the catalyst surface. 

In addition, MCD6 achieved the lowest O_3_ emission concentration at different discharge powers. The reason might be that the increase of Mn doping ratio affected the structure of MCDx and increased the oxygen vacancies in the catalyst. Therefore, MCD6 had more excellent O_3_ decomposition ability and promoted O_3_ to generate more active particles to degrade toluene, which also corresponded to the best toluene catalytic performance of MCD6.

#### 2.2.2. Catalyst Stability Analysis

**Figure 9:**
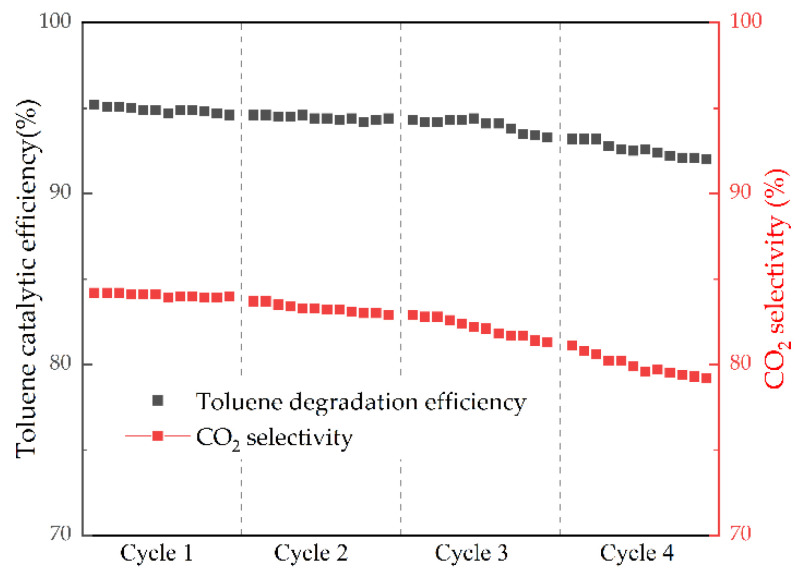
Catalytic stability test for MCD6.

In the reaction of toluene catalyzed by NTP and MCDx, due to the incomplete oxidation of toluene, some organic by-products might be produced. With the extension of reaction time, organic by-products would accumulate on the surface of the catalyst and cover the active sites, which would reduce the activity of the catalyst and affect the catalytic effect. In this experiment, MCD6, exhibiting the best synergistic catalytic performance with NTP, was selected to test its durability. The input power of the test condition was 17 W, and the remaining experimental parameters were consistent with the previous experiments. The test results are shown in Figure 9. 

It can be seen from the curves that after four cycles of 6 h stability tests, the toluene catalytic efficiency of MCD6 remained basically unchanged, decreasing from 95.2% to 92%. This indicated MCD6 could still maintain high catalytic performance after a long catalytic time. Meanwhile, the CO_2_ selectivity exhibited a decrease of about 5%, from 84.2% to 79%, and gradually stabilized, showing relatively good stability. 

**Figure 10:**
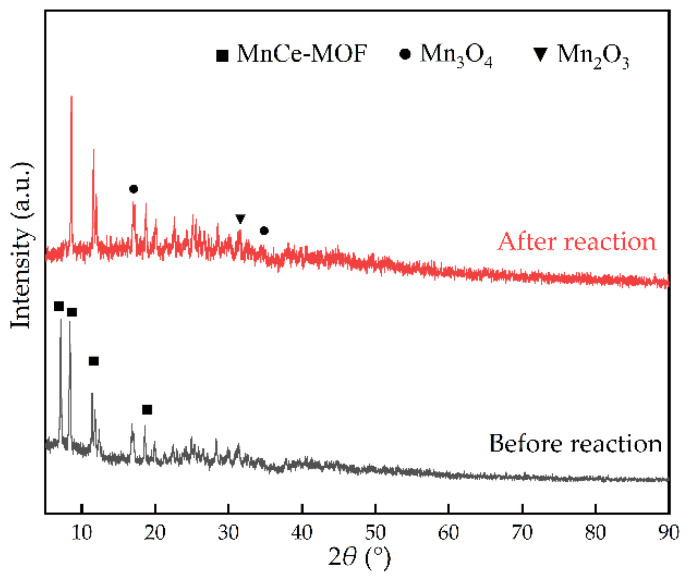
PXRD patterns of MCD6 before and after reaction.

In addition, the used MCD6 in NTP catalytic reaction was characterized by PXRD to determine whether the crystal structure of the material changed before and after the reaction. As shown in Figure 10, after four cycles of 6 h stability tests, it was found that the characteristic diffraction peaks of the material before and after the reaction changed slightly, which mainly appeared in the doping peak of Mn at 2*θ* = 7.2°, but that the typical MOF peaks did not change significantly, indicating that the NTP discharge did not change the crystal structure of the catalyst. The synergistic catalytic toluene system of NTP and MCDx showed high efficiency and stability, revealing good research and application prospects.

#### 2.2.3. Mechanism Analysis

The non-thermal plasma synergistic catalytic treatment of toluene was a complex physical and chemical process [1,9,24]. When MOFs and plasma interacted together, more complex reaction processes and active particles participated in the degradation of toluene. In addition, the excellent adsorption performance of MOFs could capture toluene in its porous structure, prolong the residence time of toluene in the reaction system, increase the collision probability between molecules, and thus improve the degradation rate.

According to related studies [10,25,26], the reaction mechanism of the non-thermal plasma synergistic catalytic treatment of toluene mainly had two parts: gas phase reaction and catalyst surface reaction. The gas phase reaction process was further divided into two pathways, one was the device discharge generated high-energy electrons, with which toluene would collide and break into small molecule organics; the other was that parts of high-energy electrons collided with the background gas to produce active particles with strong oxidation ability, which reacted with toluene or intermediates and finally decomposed to H_2_O and CO_2_ [13,16,20]. 

The catalyst surface reaction was the oxidation reaction of toluene or other intermediate products with active sites on the catalyst surface. The oxidation reaction of toluene consumed the catalyst surface active oxygen and required a constant replenishment of lattice oxygen. Additionally, the interaction between manganese oxides in the catalyst provided additional catalytic active centers for the decomposition of toluene and fixed O_2_ on the catalyst surface via a simple interconversion of Mn^2+^ to Mn^3+^, generating reactive oxygen species, which eventually led to the formation of H_2_O and CO_2_ [15,28].

## 3. Materials and Methods

### 3.1. Material Preparation Method

#### 3.1.1. Experimental Materials

The metal elements in the Mn/Ce bimetal organic frameworks were obtained from the precursors of their respective metal salts, MnCl_2_ 4H_2_O (analytical purity 99.0%, Macklin, Shanghai, China), Ce(NO_3_)_3_ 6H_2_O (analytical purity 99.5%, Macklin, Shanghai, China). The ligand of the bimetallic organic framework material was 2,5-dihydroxyterephthalic acid (analytical purity 98.0%, Macklin, Shanghai, China). The washing solvent in the catalyst preparation process mainly used N,N-dimethylformamide (DMF, analytical grade 99.5%, Sinopharm Group, Shanghai, China), ethanol (75.0%, Sinopharm Group, Shanghai, China), deionized water (analytical grade, Sinopharm Group, Shanghai, China)

#### 3.1.2. Preparation of MCDx

In this experiment, MCDx (x = 1, 2, 4, 6) was prepared by a typical solvothermal method [41]. The total amount of MnCl_2_ 4H_2_O and Ce(NO_3_)_3_·6H_2_O was 21 mmol, and the molar ratio was 1:1, which formed a solid mixture with 7 mmol of 2,5-dihydroxyterephthalic acid (DHTP). The mixture was added to a mixed solution consisting of N,N-dimethylformamide (DMF), ethanol, and deionized water (volume ratio 15:1:1), heated at a constant temperature of 70 °C, and continuously stirred in ultrasonic waves for 30 min until completely dissolved. After the stirring was complete, the solution was transferred to a 200 mL high-pressure reactor lined with polytetrafluoroethylene material, and placed in a bench-top drying oven at 160 °C for 18 h. After the reactor was naturally cooled to room temperature, the supernatant was removed, and the obtained solid product was filtered and washed three times with ethanol and deionized water. After each washing, it was centrifuged at 4000 r/min for 30 min. Finally, the obtained solid product was placed in a vacuum drying oven at 90 °C and a vacuum pressure of 0.08 MPa for 12 h. The final sample was recorded as MCD1. MCDx with different Mn doping amount were prepared by changing the molar ratio of MnCl_2_ 4H_2_O and Ce(NO_3_)_3_·6H_2_O, where x represented the molar ratio of Mn to Ce at x: 1 (x = 1, 2, 4, 6).

### 3.2. Characterization Methods

PXRD was measured using an X-ray diffraction analyzer produced by Rigaku Corporation of Japan. SEM experiments were carried out using JSM-7610F scanning electron microscope combined with EDS produced by JEOL to analyze the morphology and structure of the catalyst surface. The Thermo ESCALAB 250XI was used for XPS analysis, with the X-ray source at 150 W, the Al K*α* radiation at 1486.6 eV, the pass energy of 46.95 eV, and binding energy precision within *±* 0.3 eV. The C 1*s* line at 284.6 eV was introduced as a reference. The N_2_ adsorption isotherms of MCDx was tested by a Maxon Tristar II 3020 micropore-size analyzer (Micromeritics, Norcross, GA, USA). The samples were vacuum degassed at 350 °C for 10 h and then the surface areas and the pore-size distributions were measured according to the BET plot linear portion. FTIR experiments were carried out by Tensor27 Fourier transform infrared analyzer produced by Burker Company, Germany to analyze the phase species and surface groups of the catalyst. TGA experiments were tested using a microcomputer differential thermal balance HTC-4 produced by Beijing Hengjiu to test the thermal stability of the catalysts.

### 3.3. NTP and MCDx Synergistic Catalysis of Toluene

The NTP reactor used in this experiment was a laboratory-made dielectric barrier discharge reactor [42]. The reactor body was made of a cylindrical quartz tube with an inner diameter of 8 mm, a wall thickness of 1.2 mm, and a length of 160 mm. The quartz tube acted as a blocking medium during the discharge process. Copper mesh length 50 mm wrapped in the center of the outer surface of quartz tube, as grounding electrode. A copper rod with a diameter of 2.8 mm and a length of 350 mm was placed in the center of the quartz tube as a high voltage electrode and secured with sealant. There was an inlet (outlet) at the end of the reactor.

The amount of MCDx catalyst was 0.5 g, which was placed downstream of the NTP discharge region, and the gas hourly space velocity (GHSV) was 30,000 h^−1^. The initial concentration of toluene was 250 ppm. Each experiment began to measure after the concentration of toluene at the inlet and outlet was stable. The VOCs analyzer (PV6001-VOC, Hunan Rike Instrument Co., Ltd. Changsha, China) was used to detect the concentration of toluene and the gas analyzer (PTM600-5, Shanghai Kuanzhi Electronic Technology Co., Ltd. Shanghai, China) was used to detect the concentration of by-products CO, CO_2_, NO, NO_2_ and O_3_.

The input power of NTP power supply was set at 10 W, 11.5 W, 13 W, 15 W and 17 W, and the discharge power was measured by Lissajous method. The high-voltage probe was connected to the high-voltage electrode of the reactor, and the passive probe was connected between the reactor and the sampling capacitor. The two voltage signals were input into the oscilloscope to obtain a Lissajous figure. The discharge power *P* of the plasma in one cycle could be obtained by integrating the area of the figure:

(4) $$P=f\times C_{m}\times S$$

where *f* was the discharge frequency (Hz) and *S* was the area of the Lissajous pattern.

The specific input energy (*SIE*) of NTP discharge could be obtained by discharge power:

(5) $$SIE\left(J/L\right)=\frac{P}{Q}\times 60$$

where *P* was the discharge power (W) and *Q* was the gas flow rate (L·min^−1^).

### 3.4. Performance Evaluation Method

The performance evaluation mainly considered the following parameters: toluene catalytic efficiency, energy efficiency and CO_2_ selectivity, calculated as following:

(6) $$\eta_{toluene}(\%)=\frac{{\left[toluene\right]}_{in}-{\left[toluene\right]}_{out}}{{\left[toluene\right]}_{in}}\times 100\%$$

(7) $$\eta_{energy}(g/kWh)=\frac{{\left[toluene\right]}_{in}\times \eta_{toluene}\times M\times 0.15}{SIE}$$

(8) $$S_{CO_{2}}(\%)=\frac{{\left[CO_{2}\right]}_{out}}{7\times ({\left[toluene\right]}_{in}-{\left[toluene\right]}_{out})}\times 100\%$$

where [*toluene*]*_in_* and [*toluene*]*_out_* were the toluene concentrations at the inlet and outlet of the NTP reactor, respectively; [*CO*_2_]*_out_* was the CO_2_ concentrations at the NTP reactor outlet; *M* was the relative molecular mass of toluene. The energy efficiency referred to the mass of degraded toluene per unit of energy consumed, reflected the energy consumption of the NTP-catalyst reaction system.

## 4. Conclusions

In this experiment, a large flow of low-concentration toluene was selected as a typical industrial VOCs pollutant, and a self-made dielectric barrier discharge reactor was used to study the catalytic reaction of toluene. A series of MCDx (x = 1, 2, 4, 6) materials with different Mn: Ce molar ratios were prepared by solvothermal method. The synergetic catalytic oxidation of toluene by NTP and MCDx was studied. The effects of different input power and different MCDx catalysts on the catalytic performance of toluene were investigated.

(1) In the NTP-MCDx synergistic catalytic system, compared with the single NTP, the addition of MCDx not only significantly improved the toluene catalytic efficiency, energy efficiency and CO_2_ selectivity, but also significantly reduced the emission concentration of by-product O_3_.
(2) In each system with various MCDx, the order of toluene catalytic performance was: MCD6 > MCD4 > MCD2 > MCD1 > NTP. When the input power was 17 W, MCD6 achieved the highest toluene catalytic efficiency (95.2%) and the highest CO_2_ selectivity (81%).
(3) MCD6 with the best synergistic catalytic performance was selected for durability test. The results showed that the toluene catalytic efficiency and CO_2_ selectivity decreased by less than 5% and gradually stabilized, showing good durability, and NTP discharge did not change the structure of the catalyst.

NTP-catalyst synergistic catalytic technology has been widely studied and applied in the treatment of VOCs due to its advantages of high degradation efficiency, fast reaction speed and simple equipment. This technology is mainly used to treat large-flow and low-concentration pollutant gases at room temperature. In practical industrial applications, due to the large gas flow and fast flow rate of industrial waste gas entering the NTP reactor, the residence time of VOCs in the reactor is greatly shortened, resulting in reduced catalytic performance and increased energy consumption. Exploring new and efficient catalysts for improving degradation efficiency and energy efficiency, enhancing product selectivity and stability, and reducing by-product concentration provides guidance for large-scale industrial applications of this technology.

## Figures and Tables

**Table 1:** The quantitative analysis of valence states of the main elements.

Sample	Element	Mn^2+^/Mn^3+^	Mn^3+^/Mn^4+^	Element	Ce^3+^/Ce^4+^
MCD1	Mn 2p_3/2_	0.0342	0.6473	Ce 3d_3/2_	0.2247
MCD2	Mn 2p_3/2_	0.9125	0.6320	Ce 3d_3/2_	0.2442
MCD4	Mn 2p_3/2_	3.0948	0.2692	Ce 3d_3/2_	0.2607
MCD6	Mn 2p_3/2_	4.9645	0.1699	Ce 3d_3/2_	0.3007

## Data Availability

Not applicable.
